# Research on Saliva Secretion and Composition

**DOI:** 10.1155/2018/7406312

**Published:** 2018-06-26

**Authors:** Elsa Lamy, Fernando Capela-Silva, Asta Tvarijonaviciute

**Affiliations:** ^1^Instituto de Ciencias Agrarias e Ambientais Mediterranicas (ICAAM), Universidade de Évora, 7000-083 Évora, Portugal; ^2^Departamento de Biologia, Escola de Ciencias e Tecnologia, Universidade de Evora, 7000-671 Evora, Portugal; ^3^Interdisciplinary Laboratory of Clinical Analysis (Interlab-UMU), Regional Campus of International Excellence “Campus MareNostrum”, University of Murcia, 30100 Espinardo, Murcia, Spain

In the last two decades, saliva gained enormous attention and has become focus of a high number of studies, which resulted in a considerable progress in the knowledge about this biofluid. The technological and analytical advances, including the application of omics approaches, allowed the identification of numerous molecules in saliva, many of which are present in this fluid in proportion to blood. This permitted saliva to be seen as a fluid with potential for diagnosis of different pathologies and physiological conditions. Moreover, the noninvasive nature of its collection is an advantage over serum and reinforced the interest in saliva research.

This special issue put together high-quality research, highlighting the diversity of areas in which the study of saliva is of interest.

Ten articles, three reviews and seven original research articles, were selected for publication. The review presented by Z. Zian et al., about the use of saliva in systemic sclerosis studies, focuses on the proteomic technologies, highlighting the potentials and limitations of using saliva as a tool of diagnostic of this disease. F. Asa'ad et al. reviewed the current knowledge in saliva research applied to psoriasis, emphasising that salivary biomarkers of this disease can be valuable in the future. Finally, the review of E. Kubala et al. presented information about the diagnostic value of saliva as a research material, in the field of dental treatment. The authors concluded that more and more internists, paediatricians, pharmacologists, clinical and forensic pathologists, endocrinologists, immunologists, psychologists, and dentists are discovering the benefits offered by saliva as diagnostic tool.

Two of the original articles presented results about salivary antioxidants variations during disease treatment. One of these works, reported by A. Tvarijonaviciute et al., studied the effect of* Chamaemelum nobile *on the oxidative stress parameters and their relationship to clinical symptoms in patients with oral lichen planus. The obtained results suggest that use of* Chamaemelum nobile *could stabilize this disease, at least in medium-low severity cases. Meanwhile, K. Fejfer et al. studied oxidative stress in saliva in morbid obese individuals, before and after bariatric surgery. They observed the presence of oxidative stress-related modifications of salivary biomolecules, which although improved after bariatric surgery was not completely effective in restoring redox balance in the oral cavity.

Besides its potential in the diagnosis of oral and systemic diseases, saliva also has a major importance in food perception. Evidences show that saliva modulates oral sensations, such as astringency and/or basic tastes, or even aroma perception, suggesting that more knowledge about this biofluid composition and the factors influencing its secretion should be evaluated. Under this topic, two articles present results about the relationship between saliva and taste: (1) S. Satoh-Kuriwada et al. evaluated the effects and mechanisms of tastes on labial minor salivary gland secretion and concluded that although all basic tastes cause a gustatory-reflex secretion in minor glands, sour and umami tastes have a major effect; (2) Y. Feng et al. characterized the composition, in microorganisms and their metabolites, of the film lining the tongue, and highlighted the importance in considering the salivary microbiome in studies evaluating relationship between oral medium characteristics and taste perception.

On the other hand, a number of studies exist dealing with the effects of salivary flow influence on the analytes of interest. However, the possible effects that could result in salivary flow changes are less studied. With the aim of filling this gap of knowledge, R. H. Affoo et al. evaluated the effect of tooth brushing on salivary flow rate, comparing manual and electrical tooth brushing. The results indicate that the flow rate increases up to 5 minutes following this process. This observation was even more pronounced in older individuals.

Finally, salivary proteomics was performed in two different studies. K. T. B. Crosara et al. using proteomic approaches identified a number of interactions between amylase and other proteins present in saliva, pioneering the exploration of the vast salivary interactome. Interestingly, the authors suggest that salivary amylase may have other major functions besides carbohydrate digestion. The second article was reported by S. Lucena et al. being the only one treating nonhuman saliva. In this study, it was observed that dog saliva proteome is influenced by the breed of the animals. Moreover, acid stimulation, which is frequently used as a way of stimulating higher saliva secretion, induced changes in saliva protein composition that should be taken into account in dogs as well as other animal species and humans when designing the experiments.

Overall, studies that form the presented special issue, although coming from different fields, report the potential of saliva as a noninvasive biofluid and highlight the importance of going deep in this area.

*Elsa Lamy* *Fernando Capela-Silva* *Asta Tvarijonaviciute*

